# Abdominoscrotal Hydrocele in an Adult: The Rarest Form of Hydrocele

**DOI:** 10.7759/cureus.46934

**Published:** 2023-10-13

**Authors:** Sanjit Prasad, Vishnu S Ojha, Surya Vikram, Abhishek Kumar, Deepak Kumar

**Affiliations:** 1 Department of General Surgery, All India Institute of Medical Sciences, Patna, Patna, IND; 2 Department of General Medicine, All India Institute of Medical Sciences, Patna, Patna, IND

**Keywords:** abdominoscrotal hydrocele, hydrocele, adult, abdominal cyst, inguinoscrotal hydrocele

## Abstract

Abdominoscrotal hydrocele (ASH) represents a rare condition characterized by abdominoscrotal cystic enlargement that exhibits hourglass-shaped, fluid-filled accumulation communicating with scrotal and abdominal components on contrast-enhanced computed tomography. We present the case of a 44-year-old patient who presented with swelling in the right scrotal and abdominal regions. Upon examination, a positive cross-fluctuation was observed between the right scrotal swelling and the abdominal swelling, raising suspicions of ASH, which was subsequently confirmed radiologically. The patient underwent a right-sided sac excision and sac ligation at the deep ring performed through a right inguinal approach and subsequently experienced a smooth and uncomplicated recovery during the postoperative period. Surgical repair is the most common management approach rather than conservative management, and the use of an inguinal method over other surgical methods facilitates a lower risk of inadvertent injury to adjacent anatomical structures.

## Introduction

Hydroceles are typically characterized by an accumulation of serous fluid within the tunica vaginalis, resulting in painless scrotal swelling [1]. The least common type of hydrocele is abdominoscrotal hydrocele (ASH) [2]. ASH manifests when a hydrocele within the scrotum extends into the abdominal cavity through the inguinal canal, creating a distinctive hourglass-like configuration on contrast-enhanced computed tomography (CECT). ASH is a relatively uncommon clinical entity that primarily presents in pediatric patients. However, encountering this condition in adults is a rare occurrence [3], often challenging clinicians with diagnostic and management dilemmas. This case explores the intriguing clinical presentation, diagnostic workup, surgical intervention, and postoperative outcomes of ASH, a distinctly uncommon subtype of hydrocele, in an adult patient.

## Case presentation

**Figure 1:**
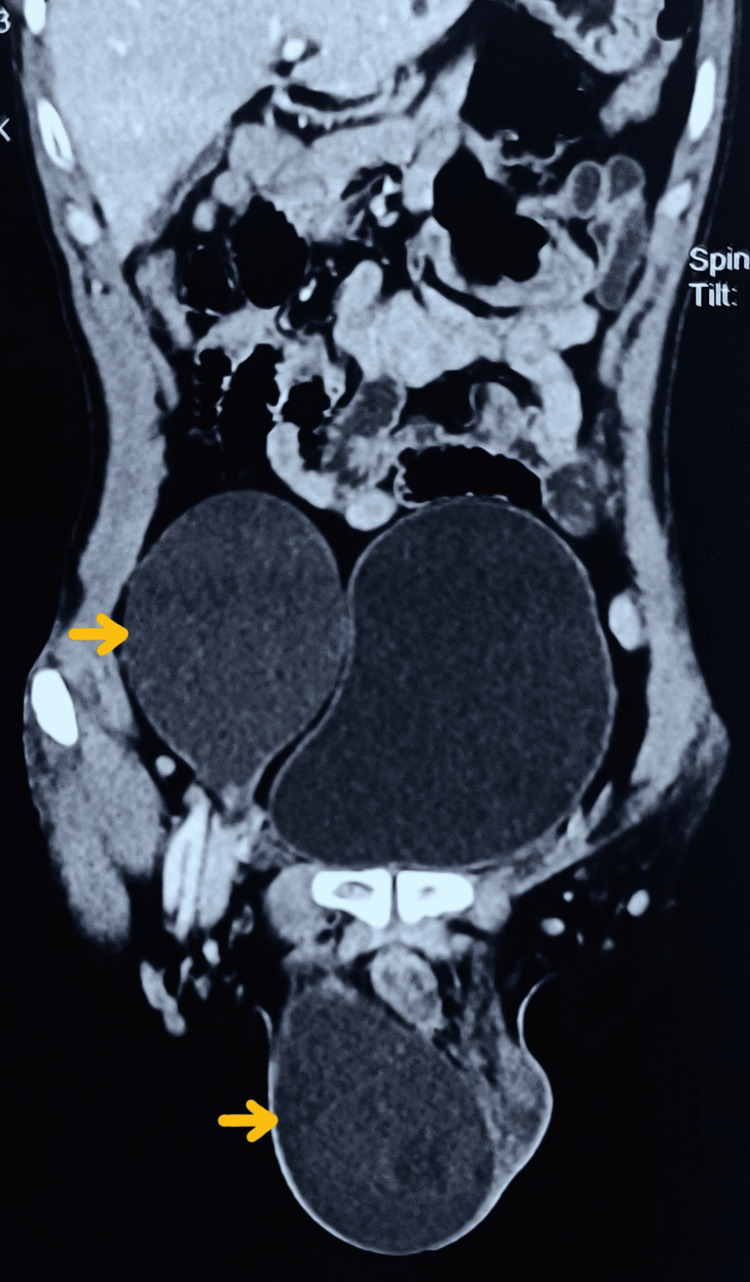
Contrast-enhanced computed tomography of the coronal section showing cystic abdominal swelling in continuity with the scrotal swelling (marked with yellow arrows).

A 44-year-old man presented with unilateral scrotal and lower abdominal swelling, along with dull aching pain and discomfort for the last six months. The patient also reported pain when coughing. There was no history of trauma or prior surgery. Upon examination, right-sided scrotal swelling and right lower abdominal swelling were identified. Both swellings were non-reducible, non-tender, transluminal, and fluctuant. Cross-fluctuation was observed with the swelling, indicating the likelihood of right-sided simple ASH (ASH-S1). ASH was not associated with any congenital anomaly or underlying disorder. The CECT of the abdomen revealed an hourglass-shaped, communicating fluid-filled accumulation that extended into the right scrotum and right lower abdomen (Figure 1).

**Figure 2:**
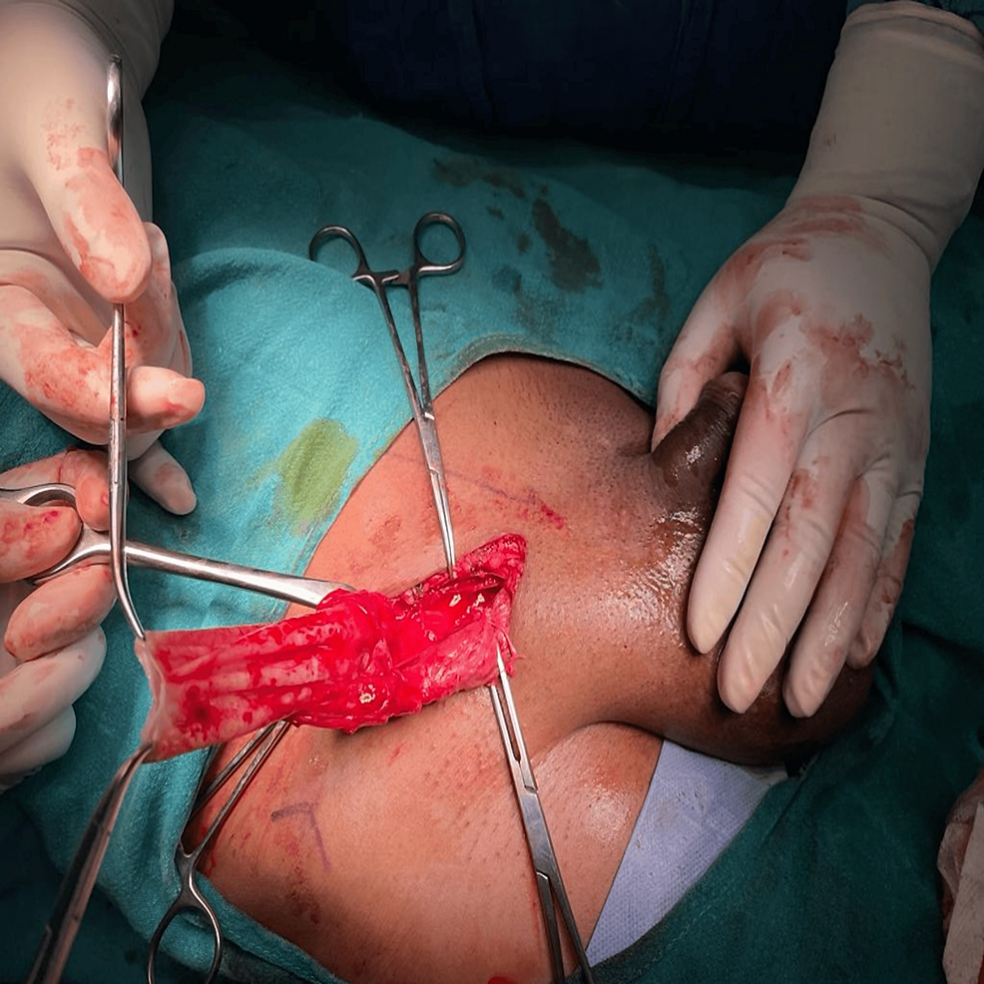
Intraoperative image of the sac approached through an inguinal incision.

During surgery, it was found that the swelling originated from the spermatic cord at the deep ring. The swelling had bilobar components: one was the abdominal part, and the other was the scrotal portion. Both components were connected by an isthmus, which was obliterated inside the inguinal canal. Each lobe measured 10 × 10 centimeters in size, containing approximately 280 mL of fluid. The abdominal component was reduced first and excised, followed by the removal of the scrotal component and ligation of the isthmus at the deep ring. The incision was exclusively inguinal, and the scrotal swelling was extracted by pulling it through the inguinal incision (Figure 2). Microscopic fluid analysis showed the presence of sterile fluid characterized by a low concentration of cellular components.

Postoperatively, the patient experienced mild pain at the operated site, which subsided with analgesics. The suture site healed well, and the patient did not have any episodes of fever during the hospital course. The patient was discharged on postoperative day three.

## Discussion

ASH was originally described by Dupuytren in 1834, and he referred to it as “hydrocoele en bissac” [4]. Typically, ASH is diagnosed in the pediatric population, but on rare occasions, it can remain asymptomatic and present in adults. The pathophysiology of ASH remains a topic of debate, with various hypotheses proposed, including Dupuytren’s hypothesis, Jacobson’s hypothesis, and Roller’s hypothesis, among others, although the most widely accepted is Jacobson’s hypothesis with modifications [3].

A rise in intraluminal pressure inside the proximal processes vaginalis is attributed by Brodman et al. to fluid buildup within the tunica vaginalis. In accordance with Laplace’s law, which stipulates that as the fluid collection’s radius grows, the internal pressure decreases, and this heightened pressure subsequently traverses the internal inguinal ring and enters the abdominal cavity, facilitating further expansion of the ASH [5]. However, despite these hypotheses, further research is necessary to precisely determine the underlying cause.

The diagnosis of ASH can be achieved through clinical examination, wherein the identification of a lump above the inguinal ring and the observation of fluid movement between the abdomen and scrotum upon compression of the tissues provide clarity regarding the nature of the swelling [6]. Imaging techniques are used in conjunction with a clinical evaluation to make the diagnosis of ASH. The existence of cross-fluctuation across abdominal and scrotal accumulations as well as a positive transillumination test are clinical criteria for diagnosis. Ultrasonography is the preferred method for visualizing the connection between the two components, though computed tomography (CT) with contrast and magnetic resonance imaging (MRI) can also be used to make a conclusive diagnosis [7]. In our case, the diagnosis was made based on the CECT scan.

Due to the paucity of standard guidelines, the management of ASH varies across centers. Various techniques have been documented in the literature, including inguinal approaches either with or without scrotal involvement, inguinal methods combined with extraperitoneal or intraperitoneal approaches, or laparoscopic approaches [2]. In our case, the inguinal approach was preferred due to its excellent view, access to the scrotal and abdominal components, and cosmetically better outcome due to the small incision along the inguinal skin crease line. 

Some studies also report on the conservative management of ASH. However, complications due to ASH are often brought on by pressure effects on nearby structures, such as hydronephrosis or unilateral leg edema brought on by compression of the iliac vein or ureter, respectively. Thus, surgical intervention is preferred over conservative management [8-10].

## Conclusions

ASH is a rare type of hydrocele, primarily documented in a limited number of case reports. Currently, there is no established standard guideline for the management of ASH. Typically, a combination of physical examination and ultrasonography suffices for diagnosis, although additional diagnostic tools such as CT scans and MRI may also be employed. Surgical repair is the most common management approach rather than conservative management, and the use of an inguinal method over other surgical methods facilitates a lower risk of inadvertent injury to adjacent anatomical structures.
